# Early diagnosis and survival outcomes in silicosis: a retrospective cohort study of 11,809 patients in Guangdong Province, China (1956–2020)

**DOI:** 10.3389/fpubh.2025.1587161

**Published:** 2025-05-01

**Authors:** Chunyue Fan, Yuhao Wang, Jingjing Chen, Qiaoli Wei, Shijie Hu, Lihua Xia, Jiawen Huang, Weihui Liang, Lin Wu, Xudong Li

**Affiliations:** ^1^Guangdong Province Hospital for Occupational Disease Prevention and Treatment, Guangzhou, China; ^2^Guangzhou Center for Disease Control and Prevention (Guangzhou Health Supervision Institute), Guangzhou, China; ^3^Big Data and Artificial Intelligence Center, The Third Affiliated Hospital of Sun Yat-Sen University, Guangzhou, China; ^4^School of Public Health, Sun Yat-sen University, Guangzhou, China; ^5^Department of Cardiology, The Third Affiliated Hospital of Sun Yat-sen University, Guangzhou, China

**Keywords:** silicosis, early diagnosis, survival outcomes, retrospective cohort study, occupational exposure, Guangdong Province

## Abstract

**Introduction:**

Silicosis, a progressive occupational lung disease caused by silica dust exposure, remains a global public health challenge due to limited therapeutic options. Early diagnosis is hypothesized to improve survival outcomes, yet evidence linking diagnostic stage to mortality remains scarce. This study aimed to evaluate the association between early diagnosis and survival in silicosis patients and assess the impact of delayed diagnosis on mortality.

**Methods:**

A retrospective cohort study analyzed 11,809 silicosis patients diagnosed between 1956 and 2020 in Guangdong Province, China. Data were extracted from occupational disease registries, multi-sectoral databases, and provincial monitoring systems. Exclusion criteria included ambiguous diagnosis dates, pre-adolescent exposure, and missing variables. Cox proportional hazards models adjusted for covariates (sex, age, region, industry, exposure duration) were used to assess mortality risks across stages I–III. Survival curves, temporal trends, and subgroup analyses were performed.

**Results:**

Most patients (77.8%) were diagnosed at stage I, with median survival times declining sharply across stages: 27 years (stage I), 20 years (stage II), and 11 years (stage III) (*p* < 0.001). Adjusted mortality risks increased progressively: stage II (*HR* = 1.42, 95%, *CI*: 1.33–1.51) and stage III (*HR* = 2.42, 95%, *CI*: 2.17–2.70)compared to stage I. Temporal analysis revealed peak diagnoses in 1963 and the early 1980s, stabilizing post-2006. Subgroup analyses confirmed staging as an independent prognostic factor across industries and exposure durations (*p* < 0.001).

**Discussion:**

This study demonstrates that early diagnosis significantly prolongs survival in silicosis patients, with advanced stages correlating with exponentially higher mortality. The findings underscore the urgent need for systematic early screening, such as high-resolution CT, and stricter occupational health policies to reduce silica exposure. Despite limitations, including unmeasured confounders like smoking status, this research provides critical evidence to inform global strategies for mitigating silicosis through timely detection and workplace safety reforms.

## Introduction

Silicosis is a progressive and debilitating pulmonary disease resulting from the inhalation of dust containing crystalline silica, which is prevalent in both traditional industries, such as mining, foundry operations, and road construction, and modern sectors, including denim textile manufacturing and jewelry polishing (1–4). It is currently acknowledged as one of the most prevalent and severe occupational diseases globally, imposing significant economic and health burdens on both affected individuals and society at large. Globally, the incidence, prevalence and mortality of silicosis have increased by 64.6, 91.4 and 20.8%, respectively, from 1990 to 2019 (5).Consequently, silicosis has become a critical public health issue with far-reaching global implications.

Many countries around the world have witnessed substantial outbreaks of silicosis (6), and the 2017 WHO Global Burden of Disease Study underscores that silicosis continues to be a significant global occupational health hazard (7, 8). In the regional analysis conducted in 2019, the three regions with the highest prevalence of silicosis were all located in Asia. Notably, East Asia was identified as the most affected region, accounting for approximately 90% of the total number of silicosis cases (9). Additionally, Iceland exhibited the lowest age-standardized prevalence rate (ASPR), whereas China had the highest rate at 113.15 per 100,000 individuals. This was followed by North Korea, Chile, Mexico, Italy, Brazil, Palau, Albania, and Slovenia, where the estimated rates exceeded 10 per 100,000 individuals (5). Despite this recognition, silicosis screening has not been prioritized for most workers, and delayed diagnosis remains one of the primary causes of respiratory failure and mortality (6). According to data from the U.S. Centers for Disease Control and Prevention (CDC), the case-fatality rate for silicosis among men in the United States between 1968 and 2013 was 98%, with the highest fatality rate observed in the metal mining sector at 19.3% (6, 10, 11). Although there has been notable progress in reducing silicosis cases in the United States (from 0.74 per 10^6^ in 2001 to 0.39 per 10^6^ in 2010) (12), new cases continue to emerge, particularly with an increase in acutely progressive cases among younger populations aged 15–44 years (13).In India, a study published in 2016 reported that approximately 3 million miners in the country are at high risk of developing silicosis (14).In China, from 1990 to 2021, the number of silicosis cases increased from 79,075 to 171,291, representing an increase of 116.62% and the number of deaths increased from 4,837 to 6,326, an increase of 30.76% (15).the median duration of silica exposure and age at diagnosis for silicosis were 13 years and 61 years, respectively (16), with key risk factors including working age, smoking history, and cumulative silica exposure (17). Younger patients tend to exhibit shorter exposure durations but a higher likelihood of disease exacerbation, highlighting the critical need for targeted occupational health management for this demographic (18).

There is currently no effective treatment for silicosis, a prevalent occupational disease (6). Comprehensive management strategies can improve the quality of life for patients and slow disease progression (19). Therefore, early diagnosis and prevention are critical measures to enhance patient outcomes (20). Prior research has suggested that gender bias may lead to delayed diagnosis in female workers with silicosis, resulting in missed opportunities for optimal intervention (21). Furthermore, certain biomarkers, such as tumor necrosis factor-alpha (TNF-*α*) and club cell protein 16 (CC16), may serve as valuable indicators for the early detection of silicosis (22, 23), thereby facilitating clinical decision-making in early diagnosis. However, insufficient emphasis has been placed on the importance of early diagnosis and screening for silicosis. To date, there is a paucity of objective evidence linking early diagnosis with survival rates in silicosis patients, and the precise impact of early diagnosis on survival remains unclear.

The objective of this study was to establish evidence for the association between early diagnosis and survival rates, and to assess the extent to which variations in survival can be attributed to delays in diagnosis and treatment initiation. This research is intended to serve as a constructive reference for government agencies in developing screening programs aimed at the early detection of silicosis.

## Materials and methods

### Participants and study design

Our pneumoconiosis data primarily include the incidence, survival rates, mortality, and loss to follow-up of pneumoconiosis cases in Guangdong Province from 1956 to 2019. This comprehensive database was developed by the Guangdong Province Hospital for Occupational Disease Prevention and Treatment (GDHOD).

During the data collection process, fundamental data were initially extracted from the “Pneumoconiosis Case Card” by occupational disease prevention and control hospitals at the prefecture-level city level or the centers for disease control and prevention at the city and county levels. These data were sourced from either the monitoring information system for occupational diseases and health hazard factors or the occupational disease and occupational health information monitoring system within the Chinese Disease Prevention and Control Information System. Subsequently, supplementary checks and filling of missing information were conducted using data reported from multiple sources, including the “Population Death Information Registration and Management System,” public security departments (household registration management systems at all levels), human resources and social security departments (the centralized integrated information system for human resources and social security in Guangdong Province), civil affairs departments (the national minimum living guarantee information system), medical security departments (the social insurance management information system), enterprises involved in assisting investigations, and the patients themselves. Finally, after summarization, a comprehensive database was established and submitted to the Guangdong Province Hospital for Occupational Disease Prevention and Treatment, which subsequently forwarded the data to the Institute of Occupational Health and Poison Control at the Chinese Center for Disease Control and Prevention.

**Figure 1:**
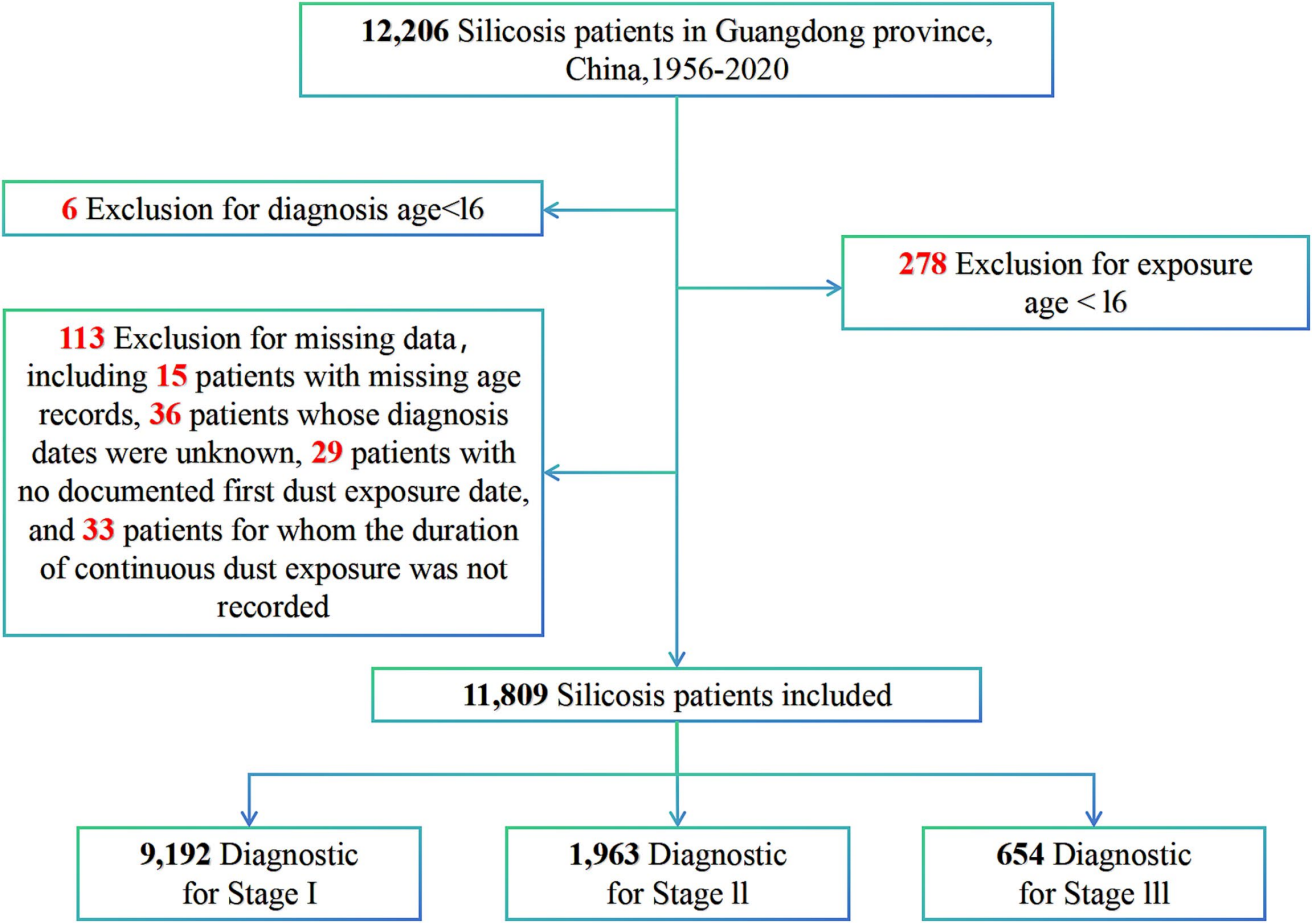
A flow chart illustrating the study participants.

A total of 12,206 cases of silicosis diagnosed between January 1956 and August 2020 were extracted from our pneumoconiosis dataset, and a retrospective cohort study was subsequently conducted. Patients were included if they were coded as J62 (pneumoconiosis due to dust containing silica), J62.8 (pneumoconiosis due to other dust containing silica), or J62.0 (pneumoconiosis due to talc) according to the International Classification of Diseases, Tenth Revision (ICD-10) (24), and if they were both employed and diagnosed in Guangdong Province. Exclusion criteria consisted of patients with an ambiguous diagnosis date, those diagnosed or exposed to silica dust before the age of 16 years, and those with missing values for the variables under investigation (overall missing rate of 0.75%). A total of 113 patients were excluded from the analysis due to incomplete data. Specifically, this included 15 patients with missing age records, 36 patients whose diagnosis dates were unknown, 29 patients with no documented first dust exposure date, and 33 patients for whom the duration of continuous dust exposure was not recorded. The final sample size for this cohort study was 11,809, and the data selection process is depicted in Figure 1.

Patients with silicosis were identified via routine annual surveillance and occupational health examinations. In China, the diagnosis of silicosis necessitates consensus among at least three radiologists in accordance with the Diagnostic Criteria for Pneumoconiosis. To ensure data consistency and comparability, standardized reporting indicators were employed, including employer information, patient name, date of birth, gender, date of diagnosis, date of first dust exposure, duration of exposure, and other relevant details. All cases of silicosis have been subject to a rigorous four-tier review process, involving the county-level Center for Disease Control and Prevention, the municipal Center for Disease Control and Prevention or the municipal Occupational Disease Prevention and Control Hospital, the provincial Occupational Disease Prevention and Control Hospital, and finally the National Center for Disease Control and Prevention, to ensure the accuracy of diagnoses. The health monitoring system, which is integrated with employer information (such as institution code, economic type, and size), facilitated dynamic observation and follow-up of reported cases. This study exclusively utilized anonymized patient information and thus did not require informed consent.

### Definition of variables

According to the “Classification and Catalogue of Occupational Diseases” in China, pneumoconiosis cases are classified into 13 types, including silicosis, coal workers’ pneumoconiosis, and graphite pneumoconiosis. In accordance with the Chinese Pneumoconiosis Diagnostic Criteria, silicosis is categorized into stages I, II, and III based on the size, density, and distribution of chest X-ray opacities, which closely aligns with the International Labor Organization (ILO) standard classification system (25, 26). The Guangdong region is subdivided into four sub-regions: the Pearl River Delta, Eastern Guangdong, Western Guangdong, and Northern Guangdong. Exposure age is stratified into four groups with a 10-year interval: <30 years, 30–39 years, 40–49 years, and ≥50 years. The cumulative dust exposure duration is defined as the total time from the onset of dust exposure until the initial diagnosis of occupational pneumoconiosis. If a patient was diagnosed with occupational silicosis but continued working in dusty environments, their actual dust exposure period terminates on the date of the first diagnosis of occupational silicosis. Dust exposure service years are further classified into three categories based on a 10-year interval: 0–10 years, 11–20 years, and >20 years (27).

In this study, industries associated with silicosis were classified into four main sectors: mining, manufacturing, utilities (including electricity, heat, gas, and water production and supply), and other industries, in accordance with the Classification of National Economy Industry (GB/T 4754–2017).

### The integration of data and the cleaning process across multiple reporting systems

This study integrates multi-source heterogeneous data via a standardized procedure. Initially, we conducted field mapping across all data sources to ensure uniformity in the names and formats of all fields. For instance, for date-related fields from different systems, we adopted the standardized “YYYY-MM-DD” format universally. During the data cleaning phase, we addressed missing value issues using advanced imputation techniques. Specifically, for time-series data, we applied linear interpolation based on adjacent time points to estimate missing values. For non-time-series data, we utilized mean imputation to maintain data integrity and consistency. To handle extreme outliers, we implemented a robust detection mechanism combining constant value checks with statistical thresholds (IQR × 3) and expert review by domain specialists. Throughout this process, original records were preserved, and cleaning operations were meticulously labeled for traceability. The entire workflow was automated using R 4.2.3, leveraging packages such as dplyr and zoo for data reconstruction and verification. Finally, the Kolmogorov–Smirnov test was employed to ensure the consistency of data distributions.

### Statistical analysis

Categorical data were presented as the number of cases (proportions) and compared using the Kaplan–Meier test. The Kaplan–Meier method was employed to plot survival curves for the null model across different diagnostic stages. The association between the diagnosis stage and silicosis-related mortality was investigated using Cox regression analysis, with covariates including sex, age at exposure, region, duration of employment, and industry. Model selection was conducted based on the Akaike Information Criterion (AIC) and the concordance index (C-index). A smaller AIC value and a larger C-index indicate a more accurate model. A sensitivity analysis of unmeasured confounders was performed using the obsSens package to further elucidate the impact of early silicosis diagnosis on patient survival. Additionally, two internal validations were carried out by adjusting the proportions of training and testing set splits to examine differences in patient survival across diagnostic stages. Statistical significance was defined as *p* < 0.05. All statistical analyses were performed using R (version 4.2.3).^1^

## Results

### Baseline characteristics of study participants

Table 1 displays the baseline characteristics of patients with silicosis stratified by diagnostic stage. This study included a total of 11,809 patients, of whom 9,192 (77.8%) were diagnosed at the early stage, 1,963 (16.6%) at the intermediate stage, and 654 (5.5%) at the late stage. The majority of patients were male, had experienced exposure prior to 1980, were younger than 40 years old at the time of exposure, had less than 20 years of occupational dust exposure, resided in mountainous regions, and worked in the mining sector. Furthermore, variations were observed across diagnostic stages in terms of age at exposure, year of exposure, duration of occupational dust exposure, geographic region, and industry type.

**Table 1:** Characteristics of silicosis patients across different diagnostic stages [*n* (%)].

Variables	Total	Stage I	Stage II	Stage III	*p*
*n*					
Sex	11,809(100.0)	9,192 (77.8)	1963(16.6)	654(5.5)	< 0.001
Male	11,616 (98.4)	9,039 (98.3)	1930 (98.3)	647 (98.9)	0.503
Female	193 (1.6)	153 (1.7)	33 (1.7)	7 (1.1)	
Exposure time					< 0.001
< 1960	4,457 (37.7)	3,480 (37.9)	818 (41.7)	159 (24.3)	
1960–1969	1967 (16.7)	1,697 (18.5)	210 (10.7)	60 (9.2)	
1970–1979	2,303 (19.5)	2016 (21.9)	220 (11.2)	67 (10.2)	
1980–1989	770 (6.5)	644 (7.0)	94 (4.8)	32 (4.9)	
1990–1999	799 (6.8)	511 (5.6)	207 (10.5)	81 (12.4)	
2000–2009	1,096 (9.3)	635 (6.9)	292 (14.9)	169 (25.8)	
2010–2020	417 (3.5)	209 (2.3)	122 (6.2)	86 (13.1)	
Exposure age, year					< 0.001
< 30	5,717 (48.4)	4,600 (50.0)	916 (46.7)	201 (30.7)	
30–39	3,328 (28.2)	2,534 (27.6)	584 (29.8)	210 (32.1)	
40–49	1955 (16.6)	1,468 (16.0)	324 (16.5)	163 (24.9)	
≥ 50	809 (6.9)	590 (6.4)	139 (7.1)	80 (12.2)	
Duration of work, year					< 0.001
0–10	4,898 (41.5)	3,526 (38.4)	989 (50.4)	383 (58.6)	
11–20	4,542 (38.5)	3,742 (40.7)	610 (31.1)	190 (29.1)	
> 20	2,369 (20.1)	1924 (20.9)	364 (18.5)	81 (12.4)	
Region					< 0.001
Mountainous Region	7,915 (67.0)	6,630 (72.1)	1,022 (52.1)	263 (40.2)	
Eastern Region	614 (5.2)	378 (4.1)	188 (9.6)	48 (7.3)	
Western Region	598 (5.1)	459 (5.0)	107 (5.5)	32 (4.9)	
Pearl River Delta	2,682 (22.7)	1725 (18.8)	646 (32.9)	311 (47.6)	
Industry					< 0.001
Mining	7,606 (64.4)	6,079 (66.1)	1,218 (62.0)	309 (47.2)	
Manufacturing	1937 (16.4)	1,427 (15.5)	355 (18.1)	155 (23.7)	
Industries	1,319 (11.2)	1,007 (11.0)	201 (10.2)	111 (17.0)	
Other	947 (8.0)	679 (7.4)	189 (9.6)	79 (12.1)	

### Diagnosis duration and all-cause mortality

**Figure 2:**
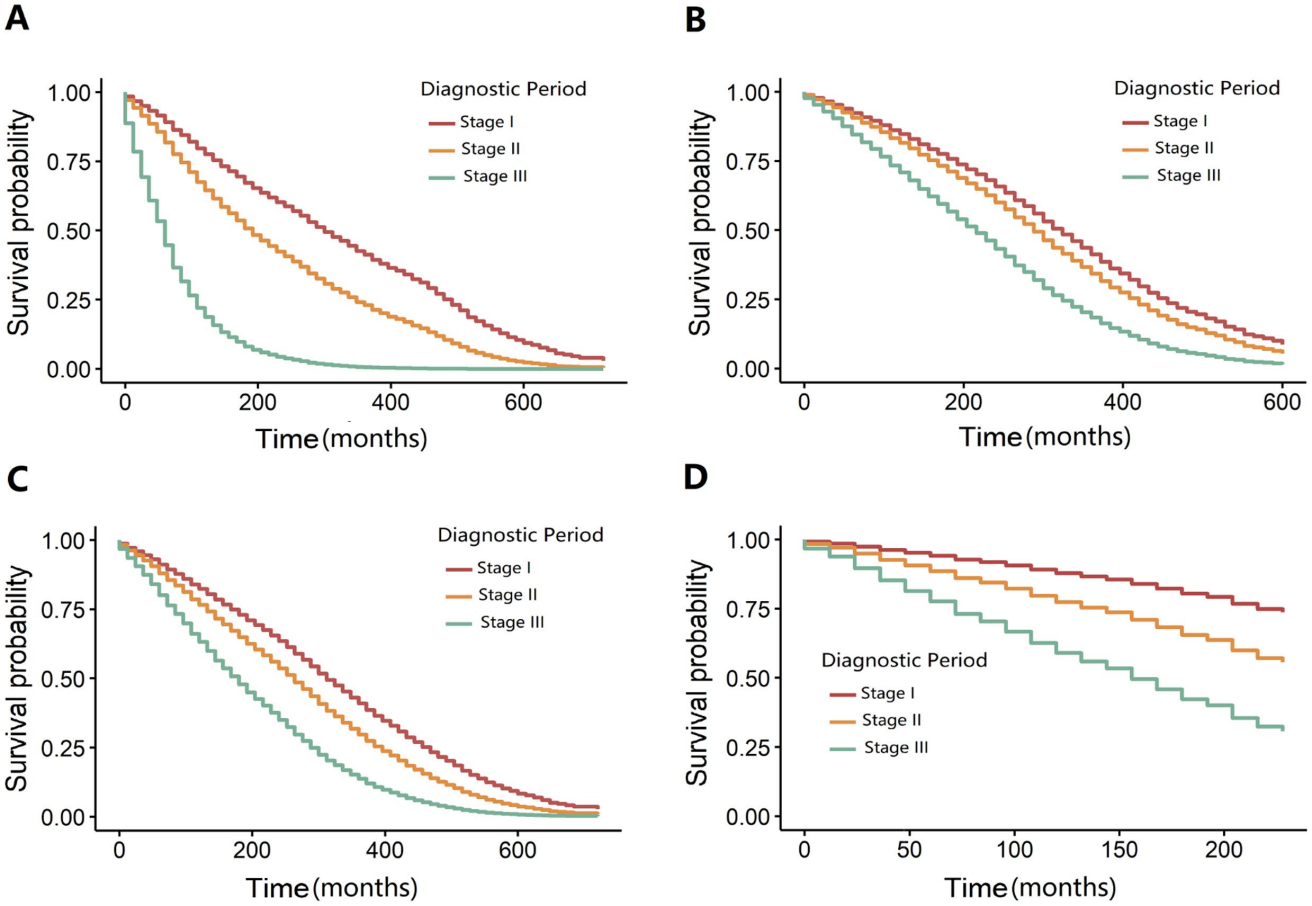
Perform a comprehensive sensitivity analysis by systematically partitioning distinct training and validation datasets. **(A)** Training set (first 20% of samples): The diagnosis year is prior to 1970. **(B)** Validation set (last 80% of samples): The diagnosis year is from 1970 onward. **(C)** Training set (first 80% of samples): The diagnosis year is prior to 2000. **(D)** Validation set (last 20% of samples): The diagnosis year is from 2000 onward.

**Figure 3:**
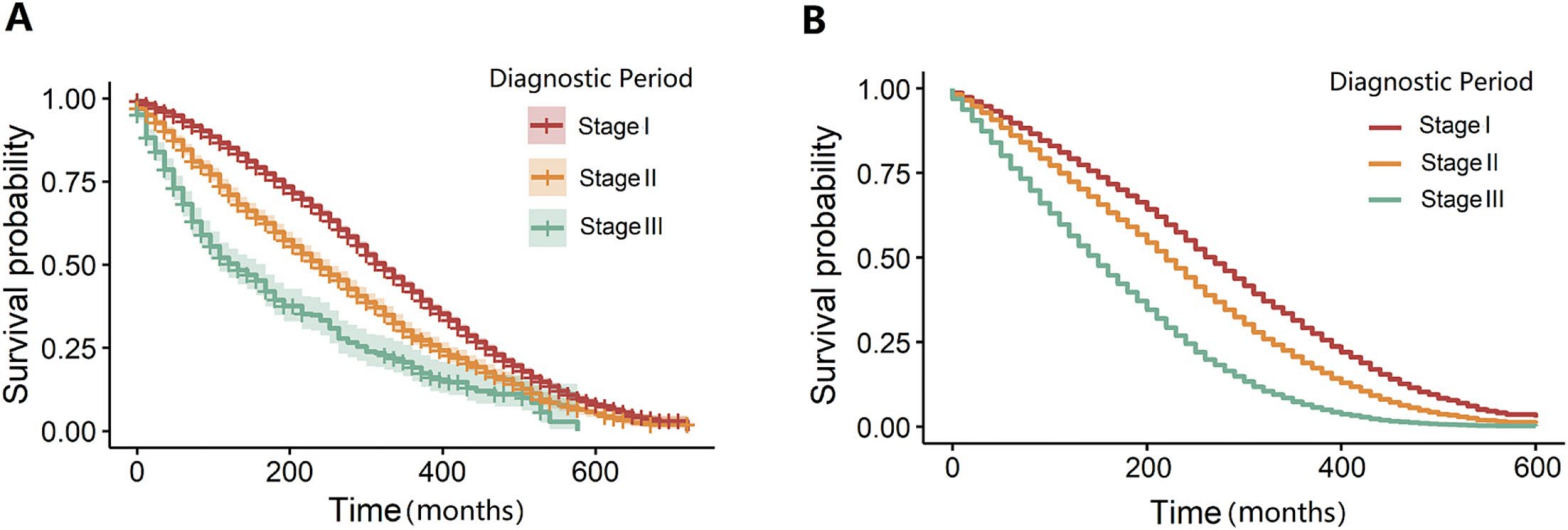
Null and fully adjusted survival curve models stratified by diagnostic period. **(A)** The adjusted survival curve model stratified by the diagnosis period is presented. **(B)** The fully adjusted survival curve model is illustrated.

The median survival time following the diagnosis of silicosis was 25 years, with substantial variability across diagnostic stages (*p* < 0.001): 27 years for stage I, 20 years for stage II, and 11 years for stage III. After comparing the Akaike Information Criterion (AIC) and C-index values among various models, Model 4 was identified as the final factor-adjusted model. The Cox proportional hazards model demonstrated that compared to stage I, a stage II diagnosis was associated with a 42% higher risk of death (Model 4: adjusted hazard ratio [*HR*] = 1.42, 95% confidence interval [*CI*] = 1.33–1.51). A stage III diagnosis corresponded to a 142% increased risk of death (Model 4: *HR* = 2.42, 95% *CI* = 2.17–2.70) relative to stage I (Table 2). Stratified analyses revealed no statistically significant association between diagnosis and mortality when the age at exposure exceeded 50 years or in the western region (Supplementary Table 1). Sensitivity analyses for unmeasured confounders corroborated the primary findings, indicating a higher risk of death for stage II and III diagnoses compared to stage I (Supplementary Table 2). Kaplan–Meier survival curves (Figure 2) illustrated differences in median survival times across diagnostic stages. Visualization of Cox regression results confirmed that both stage II and stage III diagnoses were associated with an elevated risk of death compared to stage I, both before and after adjustment (Figure 2). Furthermore, dividing the silicosis population into training and validation sets at varying proportions yielded consistent results under all four conditions, suggesting that the diagnostic stage had a statistically significant impact on silicosis-related mortality (Figure 3).

**Table 2:** Association between varying diagnostic intervals and silicosis-related mortality.

Variables	*HR* (95% *CI*)			
	Model 1^a^	Model 2^b^	Model 3^c^	Model 4^d^
Diagnostic				
Stage I	Ref.	Ref.	Ref.	Ref.
Stage II	1.45(1.36–1.54)	1.44(1.35–1.53)	1.42(1.34–1.51)	1.42(1.33–1.51)
Stage III	2.71(2.44–3.02)	2.41(2.16–2.68)	2.72(2.45–3.03)	2.42(2.17–2.70)
AIC	131,283	130686.9	130975.2	130302.1*
C-Index	0.56	0.61	0.59	0.62

a Unadjusted model.

b Adjusted sex and exposure age.

c Adjusted region, duration of work and industry.

d Adjusted sex, exposure age, region, duration of work and industry.

*According to the results of AIC, we chose Model 4 as our final model.

### Analysis of the number of cases and fatalities of silicosis

**Figure 4:**
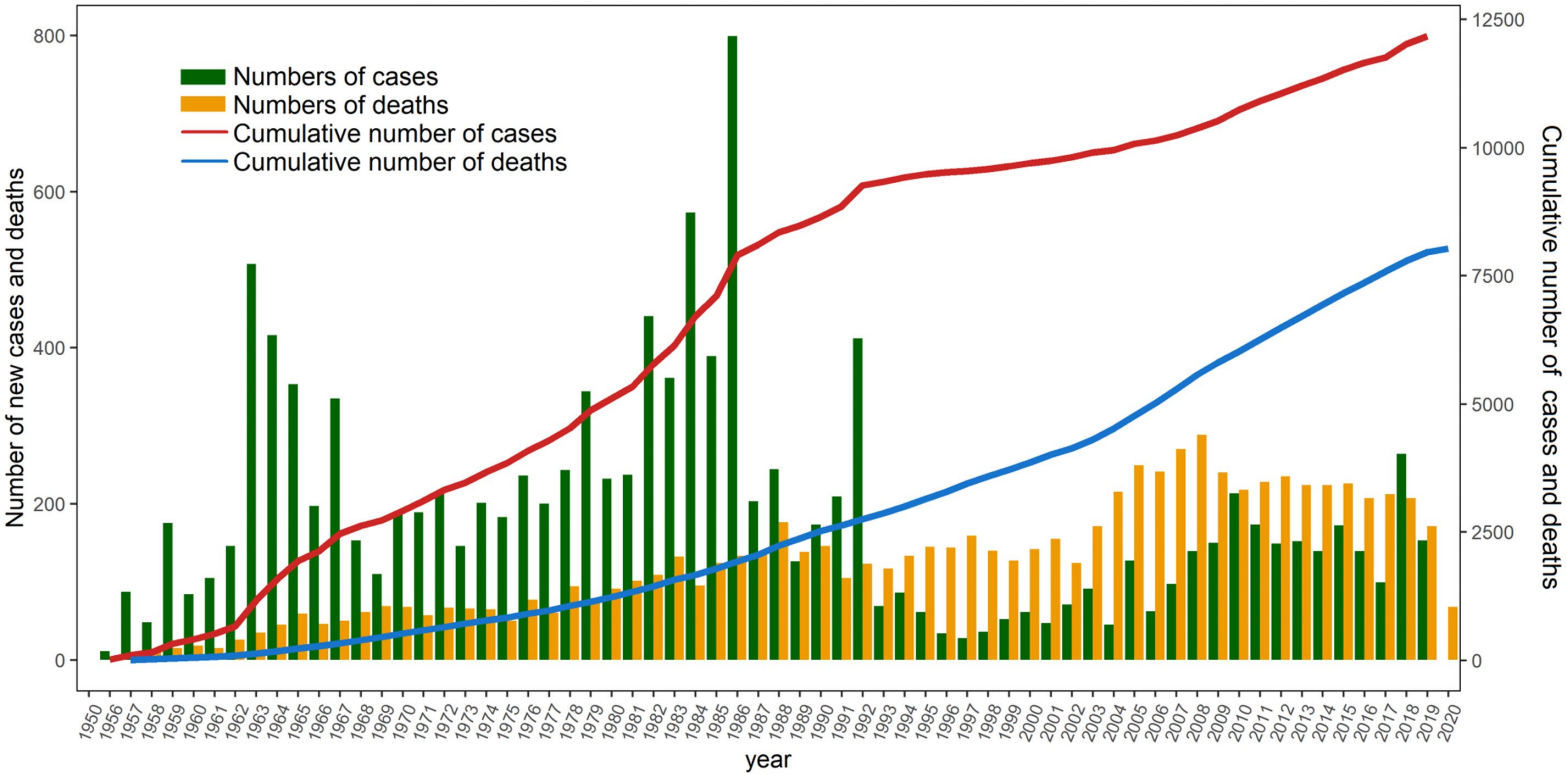
Number of cases and fatalities of silicosis in Guangdong Province, China, 1956–2020.

**Figure 5:**
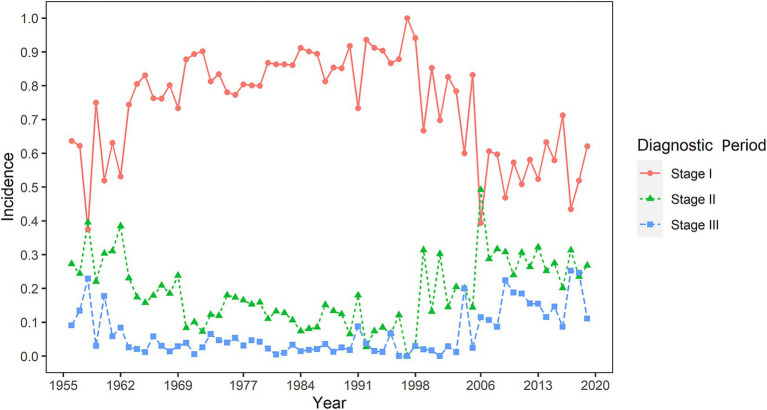
The early diagnosis rate of silicosis in Guangdong Province, China, from 1956 to the 2020s.

Figure 4 indicates that the peak number of silicosis cases occurred in 1963 and the early 1980s, whereas the peak number of deaths was observed in the late 1980s and around 2005. The gap between the cumulative number of cases and cumulative number of deaths initially widened, subsequently narrowed, and remained relatively stable with minimal fluctuations after 2011. From 1963 to 2005, the number of diagnosed stage I silicosis cases was relatively high; however, it declined and stabilized after 2006. Trends in the number of cases across diagnostic periods followed a similar pattern: an initial increase, followed by a decrease, and eventual stabilization at a low level (Figure 5).

## Discussion

The present study performed a 64-year retrospective cohort analysis of silicosis patients in Guangdong Province, China. Using a Cox proportional hazards regression model, we investigated the association between diagnostic stage and mortality among silicosis patients. The fully adjusted model demonstrated that diagnostic stage was an independent risk factor for survival in silicosis patients. Consistent results were observed in subgroup analyses, and sensitivity analyses further corroborated the robustness of these conclusions. These findings highlight the paramount importance of expediting early detection and diagnosis of silicosis to improve specialized management strategies. Although numerous studies have explored diagnostic markers associated with silicosis progression (28–32), relatively few investigations have examined the relationship between early diagnosis and patient survival. To our knowledge, this represents the largest and longest follow-up study on silicosis conducted in China and one of the first to underscore the critical significance of early diagnosis and screening for silicosis patients.

Silicosis is a chronic, irreversible respiratory disease resulting from the inhalation of respirable crystalline silica dust. To date, no effective therapeutic options are available, rendering silicosis uncontrollable (33).Over time, this disease advances to severe lung inflammation and fibrosis (34), potentially resulting in premature death or the necessity for lung transplantation (35).The Pareto diagram presented in Figure 1 indicates that the two peak periods for silicosis incidence in Guangdong Province occurred in 1963 and the early 1980s. These peaks are likely associated with the rapid industrialization during China’s early development phase, particularly in heavy industries such as coal mining, tunneling, and steelmaking (36, 37). It is estimated that between 3 and 5 million workers in Europe are exposed to crystalline silica, predominantly in the mining and construction sectors. Furthermore, exposure occurs during the processing of “engineered stones” (composite materials primarily composed of crushed quartz and marble bound with resin adhesives) and other artificial materials through activities such as grinding, polishing, drilling, and crushing (38). In regions undergoing rapid economic transformation and experiencing significant population growth, the existence of a large informal and unregulated labor force continues to substantially contribute to the burden of occupational diseases.

The Cox proportional hazards model and survival curves presented in Table 2 and Figure 4B demonstrate that, in the fully adjusted model, diagnostic staging was the most significant factor influencing survival among silicosis patients. Subgroup survival analyses detailed in Supplementary Table S1 confirm that staging remained an independent prognostic factor for survival across stratified analyses by age at exposure, years of service, and industry of employment (with the exception of age at exposure > 50 years, likely due to the long latency period of silicosis, which typically spans at least 30 years from initial exposure to death, during which competing natural mortality may overshadow silicosis-related mortality). In the United States, the National Institute for Occupational Safety and Health (NIOSH) administers the Coal Workers’ Health Surveillance Program (CWHSP), which provides coal miners with periodic chest radiographs and confidentially informs them of their pneumoconiosis status. Despite the near eradication of progressive massive fibrosis (PMF) by 1995, its prevalence among working miners increased to 3.23% by 2012. This resurgence is primarily attributed to insufficient control of dust exposures (39).

Patients diagnosed with stage III silicosis exhibited an overall mortality risk 2.42 times higher than those diagnosed at an early stage, highlighting the paramount importance of early screening for silicosis. The diagnosis of silicosis necessitates a thorough evaluation of silica dust exposure history, appropriate radiological assessments, and histopathological findings when required, in conjunction with the exclusion of other diseases that present with pulmonary nodules. The primary target population for silicosis screening comprises workers exposed to silica dust, including those employed in construction, stonework, foundries, and manufacturing sectors (6). China’s Plan for Prevention and Control of Occupational Diseases (2016–2020) explicitly emphasizes that “priority will be given to occupational silicosis and chemical poisoning, with particular attention focused on the mining, non-ferrous metal, metallurgy, and building materials industries.” For the prevention and control of silicosis, minimizing exposure to silica dust and other hazardous substances remains crucial. Additionally, one of the key strategies involves early diagnosis and screening to facilitate timely treatment and enhance survival rates. Chest computed tomography (CT) demonstrates superior efficacy compared to X-ray in detecting early-stage silicosis and exhibits an inverse correlation with pulmonary function tests (PFTs). Common CT manifestations encompass centrilobular nodules, bilateral airspace consolidation (predominantly in lower zones), calcified lymphadenopathy, and pleural thickening. Nodules and consolidation are most commonly observed in the posterior regions of the lungs (40).The promotion of HRCT in underdeveloped regions is constrained not only by equipment costs and technical thresholds but also by the infrastructure capabilities and human resource reserves of grassroots medical institutions. Importantly, technological innovation is redefining this landscape: the evolution of portable CT devices mitigates hardware deployment challenges (41), the adoption of AI-assisted diagnostic systems addresses professional talent shortages (42, 43), and the establishment of regional remote imaging collaboration networks offers a novel approach to optimizing resource allocation (44). These advancements underscore the necessity of developing a dynamic evaluation framework when formulating public health policies. This framework should acknowledge both the intrinsic limitations of CT technology and the potential of integrating emerging technological tools and innovative medical service models. By implementing adaptive strategies such as equipment-sharing mechanisms and mobile screening units, the accessibility of early diagnosis technologies for pneumoconiosis can be progressively enhanced (Figure 5).

Silicosis is a preventable occupational lung disease. The implementation of effective intervention measures, including wet cutting (which transforms silicon dust into mud), appropriate ventilation systems, and the use of high-efficiency respiratory protective equipment (rather than thin surgical masks), can safeguard workers against silica exposure. Following the introduction of stringent occupational health and safety regulations in the United States to protect high-risk workers, the incidence rate of silicosis has markedly decreased (45). The pathomechanisms of silicosis encompass the direct cytotoxic effects of silica on macrophages, activation of macrophage surface receptors, lysosomal rupture, production of reactive oxygen species (ROS), inflammasome activation, cytokine and chemokine release, cell apoptosis/pyroptosis, and lung fibrosis (46). Currently, therapeutic options for silicosis remain limited. Although lung transplantation may partially extend survival rates, there remains a significant shortage of disease-modifying drugs specifically approved for silicosis treatment. Pirfenidone and Nintedanib, which have received approval from the US Food and Drug Administration (FDA) for idiopathic pulmonary fibrosis, demonstrate potential in reducing lung inflammation, granuloma formation, and fibrosis. Tetrandrine is the only drug approved for silicosis treatment in China, and despite decades of use, its efficacy and mechanism of action remain largely unknown (47).Anti-cytokine therapies, such as the IL-1 receptor antagonist (IL-1ra) (48) and human recombinant soluble TNF receptor (49), may mitigate lung damage and fibrosis. Recently, Ramatroban, a dual antagonist of PGD2 and TXA2 receptors, has been shown to significantly alleviate silica-induced pulmonary inflammation, fibrosis, and cardiopulmonary dysfunction, thereby inhibiting the progression of silicosis (50). Gefitinib and fostamatinib effectively inhibited the levels of the phosphorylation of EGFR (p-EGFR) and SYK (p-SYK) respectively, and effectively alleviated silica-induced diffuse alveoelitis revealed by histological examination, and the levels of pro-inflammatory cytokines (such as Interleukin-1β (IL-1β), Interleukin-6 (IL-6), and Tumor necrosis factor *α* (TNF-α)) were markedly decreased in lung tissues following drug treatment (51). In addition, natural plant compounds such as sodium tanshinone II A sulfonate (STS) and kaempherol (Kae) were able to similarly inhibit silica-induced lung inflammation and fibrosis significantly (52). Early diagnosis of silicosis enables timely removal from silica-exposed environments, alveolar lavage, pharmacological treatment, and regular patient monitoring, ultimately prolonging survival time.

There are four notable limitations in our study that warrant mention. First, we did not incorporate the smoking status of patients into our analysis, despite its well-documented influence on the progression of lung diseases. Second, our analysis excluded detailed information on treatment and management strategies, which may have provided additional insights. Third, due to data constraints, we were unable to evaluate patients’ lung function using pulmonary function tests. Fourth, only “years of exposure” was utilized as a surrogate indicator for dust exposure, while the actual dust concentration or job-specific exposure levels were not taken into account. Thus, in future research, we plan to integrate dust monitoring data from occupational health records, with a particular focus on job-specific exposure levels and actual dust concentrations, to facilitate a more precise exposure assessment.

## Conclusion

This 64-year retrospective cohort study, involving 11,809 silicosis patients in Guangdong Province, China, reveals that early diagnosis markedly enhances survival outcomes. Median survival times decrease sharply from 27 years at stage I to 11 years at stage III, while mortality risks increase by 42% at stage II and 142% at stage III. These findings emphasize the critical importance of implementing systematic early screening using high-resolution computed tomography (HRCT) and enforcing stricter occupational health policies to minimize silica exposure. Although progress has been observed since 2006, persistent disparities indicate the need for targeted interventions in high-risk industries. While limitations such as unaccounted smoking status exist, this study offers valuable evidence to inform global efforts aimed at mitigating silicosis through early detection, enhanced workplace safety, and collaborative policy reforms.

## Data Availability

The original contributions presented in the study are included in the article/Supplementary material, further inquiries can be directed to the corresponding author.

## Footnotes

^1^ http://www.r-project.org/
