# Supplementary material for: Early diagnosis and survival outcomes in silicosis: a retrospective cohort study of 11,809 patients in Guangdong Province, China (1956–2020)
Source: Front Public Health. 2025 May 1;13:1587161. doi: 10.3389/fpubh.2025.1587161 (PMC12079053; doi:10.3389/fpubh.2025.1587161)
Supplement: Supplementary file 1 [file Data_Sheet_1.PDF]

Table S1. Subgroup Analysis of Survival Outcomes in Silicosis Patients Across Different Diagnostic Periods

	Group	Adjusted#	
		HR	95% *CI*
**Exposure age, year**			
<30	Stage II vs. Stage I	1.50	1.38-1.63
	Stage III vs. Stage I	3.01	2.54-3.58
30-39	Stage II vs. Stage I	1.41	1.25-1.59
	Stage III vs. Stage I	2.94	2.43-3.56
40-49	Stage II vs. Stage I	1.34	1.14-1.59
	Stage III vs. Stage I	1.89	1.48-2.41
≥50	Stage II vs. Stage I	1.03	0.80-1.34
	Stage III vs. Stage I	1.24	0.87-1.78
**Duration of work, year**			
0-10	Stage II vs. Stage I	1.50	1.37-1.64
	Stage III vs. Stage I	3.28	2.82-3.81
11-20	Stage II vs. Stage I	1.46	1.30-1.63
	Stage III vs. Stage I	1.93	1.58-2.37
> 20	Stage II vs. Stage I	1.17	1.02-1.34
	Stage III vs. Stage I	1.49	1.13-1.96
**Region**			
Mountainous Region	Stage II vs. Stage I	1.53	1.43-1.65
	Stage III vs. Stage I	2.51	2.16-2.91
Eastern Region	Stage II vs. Stage I	0.88	0.72-1.07
	Stage III vs. Stage I	2.02	1.44-2.85
Western Region	Stage II vs. Stage I	0.95	0.72-1.25
	Stage III vs. Stage I	1.53	0.96-2.43
Pearl River Delta	Stage II vs. Stage I	1.49	1.26-1.75
	Stage III vs. Stage I	2.66	2.17-3.27
**Industry**			
Mining	Stage II vs. Stage I	1.36	1.27-1.46
	Stage III vs. Stage I	2.08	1.81-2.39
Manufacturing	Stage II vs. Stage I	1.42	1.15-1.74
	Stage III vs. Stage I	3.03	2.34-3.91
Production and supply industries*	Stage II vs. Stage I	1.33	1.07-1.65
	Stage III vs. Stage I	3.04	2.18-4.24
Other	Stage II vs. Stage I	1.91	1.41-2.59
	Stage III vs. Stage I	3.26	2.13-4.99

* indicated included production and supply of electricity, heat, gas and water.

\# adjusted sex, exposure age, region, duration of work and industry.

Table S2. Sensitivity Analysis Adjusting for a Hypothetical Unmeasured Confounder with Specified Characteristics

Group	Condition	Gamma = 2				Gamma = 3				Gamma = 4			
		P0	***P1***	***HR***	***95% CI***	***P0***	***P1***	***HR***	***95% CI***	***P0***	***P1***	***HR***	***95% CI***
**Stage II vs. stage I**	Condition 1	0.2	0.1	1.51	1.42,1.60	0.2	0.1	1.57	1.48,1.66	0.2	0.1	1.63	1.54,1.72
	Condition 2	0.4	0.1	1.66	1.57,1.75	0.4	0.1	1.83	1.74,1.92	0.4	0.1	1.95	1.86,2.04
	Condition 3	0.6	0.1	1.79	1.70,1.88	0.6	0.1	2.03	1.94,2.12	0.6	0.1	2.19	2.10,2.28
	Condition 4	0.2	0.5	1.20	1.11,1.29	0.2	0.5	1.06	0.97,1.15	0.2	0.5	0.97	0.88,1.06
	Condition 5	0.4	0.5	1.35	1.26,1.44	0.4	0.5	1.31	1.22,1.40	0.4	0.5	1.29	1.20,1.38
	Condition 6	0.6	0.5	1.48	1.39,1.57	0.6	0.5	1.52	1.43,1.61	0.6	0.5	1.53	1.44,1.62
	Condition 7	0.2	0.9	0.96	0.87,1.05	0.2	0.9	0.73	0.64,0.82	0.2	0.9	0.58	0.49,0.67
	Condition 8	0.4	0.9	1.11	1.02,1.20	0.4	0.9	0.98	0.89,1.07	0.4	0.9	0.90	0.81,0.99
	Condition 9	0.6	0.9	1.25	1.16,1.34	0.6	0.9	1.18	1.09,1.27	0.6	0.9	1.14	1.05,1.23
**Stage III vs. stage I**	Condition 1	0.2	0.1	2.51	2.26,2.79	0.2	0.1	2.57	2.32,2.85	0.2	0.1	2.63	2.38,2.91
	Condition 2	0.4	0.1	2.66	2.41,2.94	0.4	0.1	2.83	2.58,3.11	0.4	0.1	2.95	2.7,3.23
	Condition 3	0.6	0.1	2.79	2.54,3.07	0.6	0.1	3.03	2.78,3.31	0.6	0.1	3.19	2.94,3.47
	Condition 4	0.2	0.5	2.20	1.95,2.48	0.2	0.5	2.06	1.81,2.34	0.2	0.5	1.97	1.72,2.25
	Condition 5	0.4	0.5	2.35	2.10,2.63	0.4	0.5	2.31	2.06,2.59	0.4	0.5	2.29	2.04,2.57

Condition 6	0.6	0.5	2.48	2.23,2.76	0.6	0.5	2.52	2.27,2.80	0.6	0.5	2.53	2.28,2.81
Condition 7	0.2	0.9	1.96	1.71,2.24	0.2	0.9	1.73	1.48,2.01	0.2	0.9	1.58	1.33,1.86
Condition 8	0.4	0.9	2.11	1.86,2.39	0.4	0.9	1.98	1.73,2.26	0.4	0.9	1.90	1.65,2.18
Condition 9	0.6	0.9	2.25	2.00,2.53	0.6	0.9	2.18	1.93,2.46	0.6	0.9	2.14	1.89,2.42
